# Targeting acidic pre-metastatic niche in lungs by pH low insertion peptide and its utility for anti-metastatic therapy

**DOI:** 10.3389/fonc.2023.1258442

**Published:** 2023-11-15

**Authors:** Toma Matsui, Yuki Toda, Haruka Sato, Rina Itagaki, Kazuya Konishi, Anna Moshnikova, Oleg A. Andreev, Shigekuni Hosogi, Yana K. Reshetnyak, Eishi Ashihara

**Affiliations:** ^1^ Laboratory of Clinical and Translational Physiology, Kyoto Pharmaceutical University, Kyoto, Japan; ^2^ Physics Department, University of Rhode Island, Kingston, RI, United States

**Keywords:** extracellular acidity, pre-metastatic niche, pH low insertion peptide, metabolic reprogramming, extracellular vesicle

## Abstract

Dysregulated extracellular pH, the universal feature of tumor, works as an evolutional force to drive dissemination of tumor cells. It is well-established that tumor acidity is associated with tumor growth and metastasis. However, the pH of pre-metastatic niche remains unclear. We hypothesized that primary tumor cells remotely prime acidity in secondary organ to achieve metastatic colonization. Herein, we demonstrated that the pH responsive probe pH Low Insertion Peptide (pHLIP) was notably accumulated in pre-metastatic lungs of 4T1.2 breast tumor-bearing mice. The pHLIP-targeted lungs showed high amounts of lactate and overexpressed glycolysis-related proteins. Pharmacological inhibition of glycolysis suppressed the lung acidification induced by 4T1.2 cancer cell culture supernatant and delayed subsequent metastatic burden of disseminated tumor cells. In the acidic lungs, pHLIP was primarily localized in alveolar type 2 cells which strongly expressed glycolysis-related proteins. 4T1.2-derived extracellular vesicles expressed some of the glycolysis-related proteins, and their administration increased pHLIP accumulation and glycolytic enhancement in lungs. pHLIP-conjugated dexamethasone effectively attenuated lung metastatic burden by disrupting pro-inflammatory response in the acidic lungs. From these results, targeting the metastasis-supporting microenvironment by pHLIP technology creates possibility to identify pre-metastatic organ and prevent metastatic recurrence.

## 1 Introduction

Acidic extracellular pH is a widely accepted physicochemical feature of tumor microenvironment (1). Increased metabolic flux and limited perfusion within tumors lead to the accumulation of acidic metabolite in its interstitial space, which contributes to tumor development. Tumor cells adapt to extracellular acidosis by altering their protein expression pattern and metabolic state (2). The acid adaptation is accompanied by acquisition of therapeutic resistance and augmented metastatic potential (3, 4). In addition to the influence on tumor cells, the environmental acidification intensifies metastatic spread by modulating stromal components such as blood vessel, extracellular matrix, and immune cells (5–7).

Despite of the significant impact of extracellular acidosis on tumor metastasis, pH condition of pre-metastatic site is unknown. Paget et al. proposed that metastasis preferentially develops in the specific site harboring a supportive microenvironment for the survival and proliferation of disseminated tumor cells (8). In some cases, primary tumor cells use their secretion factors to establish this favorable condition in the secondary site before their arrival (pre-metastatic niche formation) (9). The cellular and non-cellular components of pre-metastatic niche were investigated and identified (10, 11); however, functional role of acidity for niche development and formation has not been studied.

The family of pH Low Insertion Peptides (pHLIPs) senses extracellular acidity which triggers protonation of Asp/Glu residues and promotes peptide’s insertion into plasma membrane of acidic cells (12–14). Based on this principle, pHLIP has been developed as a marker of acidic tissue such as aggressive tumor (15). In this study, we investigated role of acidity in pre-metastatic niche development on mouse breast tumor model by employing pHLIP technology. The findings demonstrate that pre-metastatic niche in lungs is acidic, and pHLIP-drug conjugates could be developed as a targeted anti-metastatic therapy for drug delivery to the acidic niche.

## 2 Materials and methods

### 2.1 Cell lines

The murine breast cancer cell lines (4T1.2 and 66cl4) were kindly provided by Dr. Robin L. Anderson (Peter MacCallum Centre, Melbourne, Australia). 66cl4 cell line spontaneously metastasizes only to lung, while 4T1.2 cell line aggressively metastasizes to several organs (lung, axillary lymph node, and bone) (16). The extent of metastatic dissemination of 4T1.2 cancer cells into lung is larger than that of 66cl4 cancer cells. Both cell lines were maintained in Minimum Essential Medium α (MEM-α; FUJIFILM Wako Pure Chemical Corporation, Osaka, Japan) supplemented with 5% fetal bovine serum (FBS) and 1% penicillin/streptomycin at 37°C in a humidified atmosphere of 5% CO_2_ and 95% air. These cells were regularly tested negative for Mycoplasma infection using Venor^®^GeM Classic Mycoplasma Detection Kit (Minerva Biolabs, Berlin, Germany).

For *in vivo* tracking of tumor cells, pGL4.51 vector (Promega, Madison, WI, USA) harboring the luciferase reporter gene *Luc2* and neomycin resistance gene was transfected into 4T1.2 cancer cells using Lipofectamine 3000 (Invitrogen, Waltham, MA, USA). Transfected cells were selected with MEM-α containing the analog of neomycin sulfate G418 for 2 weeks and then established as 4T1.2-Luc2 cancer cells. *Luc2*-transduced 66cl4 (66cl4-Luc2) cancer cells were kind gift from Dr. Cheryl L. Jorcyk laboratory (Boise State University, Boise, ID, USA).

### 2.2 Preparation of pHLIP-conjugated agents

pHLIP peptide (ACDDQNPWRAYLDLLFPTDTLLLDLLWA) was conjugated with Alexa Fluor (AF) 647-maleimide or AF750-maleimide (Life Technologies, Carlsbad, CA, USA) in dimethylsulfoxide (DMSO) at ratios of 1:1 and incubated at room temperature (RT) for 2-4 h until the conjugation was completed. Sodium phosphate buffer (100 mM) containing 150 mM NaCl at pH 6.7 saturated with argon was added to the reaction mix (1/10 of the total volume). The reaction progress was monitored by reversed phase high-performance liquid chromatography using Zorbax SB-C18 and Zorbax SB-C8 columns (4.6 × 250 mm, 5 μm; Agilent Technology, Santa Clara, CA, USA). For synthesis of pHLIP-conjugated dexamethasone (pHLIP-Dex), the pHLIP peptide was mixed with modified dexamethasone (Iris Biotech GmbH, Marktredwitz Germany) in DMSO at a molar ratio 1:1. Sodium phosphate buffer (100 mM) containing 150 mM NaCl at pH 7.4 saturated with argon was added to the reaction mix (1/10 of the total volume), followed by incubation of the reaction mixture for 2 h at RT. All constructs were purified at Zorbax SB-C18 columns (9.4 x 250 mm, 5 µm; Agilent Technologies) and lyophilized. The constructs were characterized by surface-enhanced laser desorption/ionization time-of-flight mass spectrometry and by analytical reversed phase high-performance liquid chromatography. Each concentration of the constructs was determined by absorbance using the following molar extinction coefficients: *ε*_651 =_ 270,000 M^−1^cm^−1^ for AF647-conjugated pHLIP (AF647-pHLIP), *ε*_753 =_ 290,000 M^−1^cm^−1^ for AF750-conjugated pHLIP (AF750-pHLIP), and *ε*_280 =_ 12,660 for pHLIP-Dex.

### 2.3 Generation of orthotopic breast tumor mouse model

All animal experiments complied with ethical regulations were approved by the committee on the Ethics of Animal Research of Kyoto Pharmaceutical University (approval number: CTPH-20-006, A22-066, and A23-006). 4T1.2-Luc2 or 66cl4-Luc2 cancer cells (100,000 cells) were suspended in 0.1 mL of phosphate buffer saline (PBS) and implanted into fourth mammary fat pad of syngeneic BALB/cCrSlc mice (female, 7 weeks old) purchased from Shimizu Laboratory Supplies (Kyoto, Japan). Growth of primary tumor and metastatic burden were monitored by bioluminescence imaging. Mice were intraperitoneally injected with D-luciferin (150 mg/kg, FUJIFILM) prior to imaging by IVIS Lumina XRMS SeriesIII (PerkinElmer, Waltham, MA, USA). Bioluminescence was analyzed by Living image software v4.7.4 (PerkinElmer). At several time points from cell’s implantation, mice were sacrificed, and tissues/organs were resected for subsequent analyses. Primary tumor was weighed immediately after resection.

### 2.4 Preparation of cancer cell-conditioned media and *in vivo* pre-metastatic niche formation

4T1.2 or 66cl4 cancer cells were cultured from 30,000 cells/mL MEM-α containing 5% FBS for 48 h. After washing with PBS, cells were grown in serum-free MEM-α for additional 24 h. Collected cell-conditioned medium was centrifuged at 2,000 g for 20 min and then at 10,000 g for 30 min to remove dead cells and debris. The supernatant was filtered (pore size: 0.22 μm) and aliquoted. Serum-free MEM-α was used as control media. Female BALB/cCrSlc mice (7 weeks old) were intraperitoneally administered with 0.3 mL of either cancer cell-conditioned media or control media every day for 21 days. At day 22^nd^ after initiation of the treatment, lungs were harvested for *ex vivo*/*in vitro* analyses.

### 2.5 Drug treatment and generation of experimental lung metastasis model

To inhibit lung acidification, mice were treated with sodium oxamate (oxamate, 300 mg/kg; FUJIFILM) dissolved in either 4T1.2 cancer cell-cultured supernatant (4T1.2-CM) or MEM-α by single intraperitoneal injection every day for 21 days. To downregulate inflammatory cytokine levels in acidic lungs, pHLIP-Dex (4 mg/kg) was intraperitoneally injected once a day for 7 days. The daily treatment of pHLIP-Dex was started following the administration of 4T1.2-CM or MEM-α for 21 consecutive days.

At the day following completion of the pharmacological treatments, 50,000 of 66cl4-Luc2 cancer cells were injected into tail vein. Metastatic colonization in lungs was detected by bioluminescence imaging. At least 3 mice were analyzed at the same time. After luciferin injection, imaging was immediately started, and data were acquired at 5 min intervals. Black sheet was used to cover bottom part of mice to prevent filtering of the bioluminescence from tumor cells at injection site. Body weight was measured twice per week, and mice were euthanized at humane endpoint when animals showed signs of disease progression with a decrease in body weight of 20%.

### 2.6 *Ex vivo* imaging for organ biodistribution of pHLIP

Mice were intraperitoneally administered with a single dose of AF750-pHLIP (20 μM pHLIP/0.1 mL PBS) 24 h before sacrifice. Excised organs/tissues were rinsed with PBS and then imaged for their fluorescence derived from AF750-pHLIP by Lumina XRMS SeriesIII. Acquired fluorescence data were quantified by Living image software.

### 2.7 Tissue dissociation for cell isolation and analysis

Lungs were perfused with 10 mL PBS from right ventricle before surgical resection. Isolated lung lobes were minced into small species, followed by incubation in Roswell Park Memorial Institute medium (FUJIFILM) including 0.2 mg/mL collagenase type IV (Sigma-Aldrich, St. Louis, MO, USA) and 25 μg/mL DNase I (Roche Diagnostics, Basel, Switzerland) for 30 min at 37°C. Digested cells were filtered by 70 μm nylon filters (Greiner Bio-One, Kremsmünster, Austria). The filtered cells were centrifuged at 350 g for 5 min, and resulting pellet was resuspended in 1 mL of lysis buffer (BD Bioscience, Becton Drive Franklin Lakes, NJ, USA). After incubation for 1 min at RT, 30 mL of PBS with 2% FBS was slowly added into the incubation tube, followed by centrifugation at 350 g for 5 min. The cell pellet was resuspended in PBS with 2% FBS to use for flow cytometric analysis or further purification.

For isolation of alveolar type 2 (AT2) cells and fibroblasts, lung dissociation was performed according to previously reported protocol with some modifications (17). In brief, following transcardiac perfusion with PBS, 2 mL dispase solution (2.0 U/mL; Sigma) was instilled through intubated catheter. To avoid leakage of dispase, 0.5 mL of low melting point agarose (2% in PBS) were subsequently instilled, and then the lungs were immediately covered with ice for 2 min. After isolation of lungs from thoracic cavity, each lobe was incubated in Hank’s Balanced Salt Solution (FUJIFILM) supplemented with 1.0 U/mL dispase for 45 min at RT. Digested lobes were transferred into Dulbecco’s Modified Eagle Medium (Nacalai Tesque, Kyoto, Japan) containing 25 mM 4-(2-hydroxyethyl)-1-piperazineethanesulfonic acid, 10% FBS, and 100 U/mL DNase I. After mincing lobes into small species using scissor, the dissociated lung cells were left for 5 min at RT and then sequentially passed through 40- and 70-μm nylon filters (Greiner). After centrifugation (350 g, 5 min), lysis buffer was used to remove red blood cells. Finally, collected cells were resuspended in PBS with 2% FBS as a single cell suspension.

### 2.8 Clonogenic assay

For detection of micrometastasis, lung lobes were enzymatically dissociated according to AT2 isolation protocol as described above and resuspended in MEM-α containing 60 μM 6-thioguanine (FUJIFILM). The suspensions were plated in 6 well plate and cultured for 14 days. Colonies selected by 6-thioguanine were fixed by methanol and stained with 0.03% methylene blue (Nacalai).

### 2.9 Flow cytometric analysis and cell sorting

Lung-derived cells were blocked by anti-CD16/CD32 antibody (1:1000 dilution with PBS; eBioscience, San Diego, CA, USA) for 10 min at RT and stained at 4°C for 10 min with primary antibodies listed in **Table S1**. After washing out the antibodies, cells were incubated with AF647-conjugated streptavidin (1:500 dilution with PBS; Jackson ImmunoResearch, West Grove, PA, USA) at 4°C for 10 min. Dead cells were stained by 5 μg/mL propidium iodide (FUJIFILM) immediately before sorting. All cell sorting studies were carried out using BD FACSJazz™ (BD). Data were analyzed by Flowjo software v10.4.2. (BD).

For cytometric bead assay, 20-30 mg of lung tissue was suspended in 0.3 mL of protein extraction buffer which contained 100 mM tris(hydroxymethyl)aminomethane (pH 7.4), 150 mM NaCl, 1 mM ethylene glycol tetraacetic acid, 1 mM ethylenediaminetetraacetic acid, 1% Triton^®^ X-100 (Nacalai), 0.5% sodium deoxycholate, 1x protease inhibitor cocktail (Sigma), 40 mM sodium fluoride, and 2 mM sodium orthovanadate. Bioruptor UCD-250 (BM Equipment, Tokyo, Japan) was used to sonicate the lysate. Sonication cycles and on/off-times were set to 7 cycles and 30 s, respectively. The sonicated sample was centrifuged at 9000 g for 10 min, and resulting supernatant was used for assays. Interleukin (IL)-6 and tumor necrosis factor (TNF)-α in the samples were detected using BD Cytometric Bead Array (CBA) System with following reagents: Mouse IL-6 Flex Set, Mouse TNF Flex Set, Mouse/Rat Soluble Protein Master Buffer Kit (all from BD). CBA beads conjugated with anti-IL-6 or anti-TNF-α antibodies have different fluorescent intensity at two different channels for allophycocyanin and allophycocyanin-cyanine7. Therefore, the two types of beads can be distinguished in flow cytometry. After incubation with phycoerythrin-conjugated detection antibody, each analyte captured on beads was simultaneously measured as mean fluorescent intensity of detection antibody on a BD LSRFortessa™ X-20 (BD). Acquired data was analyzed for individual cytokine concentration based on calibration curve using Flowjo software v10.4.2. Finally, cytokine amounts were normalized by protein concentration of individual samples measured by BCA Protein assay Kit (Thermo Fisher Scientific, Waltham, MA, USA).

### 2.10 Immunocytochemistry

4T1.2-Luc2 cancer cells or isolated CD326^+^ cells were cytospun on glass slide at 500 rpm for 5 min using CYTOSPIN 4 (fisher scientific, Waltham, MA, USA). Dried for 5 min at RT, the glass slide was washed with PBS and then fixed in 4% paraformaldehyde (PFA) for 5 min at RT. Fixed cells were incubated with primary antibodies in 0.1 M PBS including 3% Triton^®^ X-100 overnight at 4°C, followed by treatment with fluorescent dye-labelled secondary antibodies and Hoechst33342 (1:5000; Thermo) for 2 h at RT. The primary and secondary antibodies are listed in **Table S1**. The immune-stained cells were mounted on glass slides with VECTASHIELD Mounting Medium (Vector Laboratories, Newark, NJ, USA), and images of fluorescence were obtained using an LSM800 laser confocal microscope (ZEISS, Oberkochen, Germany). Data was analyzed by ZEN 3.0 (ZEISS).

### 2.11 Immunohistochemistry

Twenty-four hours before sacrifice, mice were intraperitoneally injected with 0.1 mL of AF647-pHLIP (20 μM in PBS). After perfusing by PBS, lungs were harvested and then fixed with 4% PFA at 4°C for 24 h prior to dehydration with 30% sucrose solution for 3 days. Lungs were embedded in Optimal Cutting Temperature Compound (Sakura Finetek, Tokyo, Japan) and then immediately frozen at -20°C. Embedded lung tissues were sectioned at a thickness of 10 μm using a cryostat (Leica CM1950; Leica Biosystems, Nussloch, Germany). Lung sections were incubated with the primary antibodies (in 0.1 M PBS including 3% Triton^®^ X-100, overnight, 4°C) listed in **Table S1**. After washing, sections were stained with fluorescent dye-labeled secondary antibodies (**Table S1**) and Hoechest33342 (1:5000). Stained sections were mounted on glass slides with VECTASHIELD Mounting Medium. Images were captured using a LSM800 and processed by ZEN 3.0. The fluorescence data was quantified using Fiji software v1.53t.

### 2.12 Isolation, characterization, and administration of extracellular vesicles (EVs)

To avoid contaminating FBS-derived EVs, breast cancer cell-derived EVs were isolated from 4T1.2-CM or 66cl4-CM by step-wised (ultra)centrifugation (2,000 g, 20 min; 10,000 g, 30 min; 100,000 g, 2 h) (18). Final pellet was washed with PBS and then centrifuged at 100,000 g for 2 h, which was repeated until losing red color of MEM-α. EV suspension (in PBS) was aliquoted according to its protein concentration measured using Micro BCA Protein assay Kit (Thermo).

Size distribution of EVs was measured by dynamic light scattering (DLS) at 4°C using a Zetasizer Nano instrument (Malvern Instruments, Malvern, WR, UK). EV morphology was analyzed by Nanoscope IIIa Tapping Mode AFM (Veeco, Plainview, NY, USA) with a single–crystal microcantilever (OMCLAC160TS-R3, Olympus, Tokyo, Japan) (18). Sample (10 μL) was spotted onto freshly cleaved mica sheets (Nilaco, Tokyo, Japan), incubated for 10 min at RT, rinsed with 20 μL of deionized water, dried in air, and imaged in tapping mode at 0.5 Hz scan rate in air.

BALB/c mice (female, 7 weeks) were intraperitoneally administrated aliquot of 4T1.2-EVs (10 μg proteins/0.1 mL PBS) per injection every day for 21 days. PBS was used as control. For *in vitro*/*ex vivo* analyses, mouse lungs were harvested at the day following the completion of EV administration.

### 2.13 Lactate assay

To prevent lactate degradation by endogenous lactate dehydrogenase, lungs were rapidly frozen by liquid nitrogen immediately after resection. Lung tissue pieces (25-30 mg) placed in 0.3 mL 0.1% Triton^®^ X-100 were homogenized by Bioruptor II (TYPE12, BM Equipment, Tokyo, Japan) for 30 min. After centrifugation (8,000 g, 5 min), supernatant was filtered by Amicon Ultra-0.5ml centrifugal filter unit with molecular weight cut-off of 10 kDa (MERK, Darmstadt, Germany) at 12,000 g for 10 min for separation of lactate and lactate dehydrogenase. Lactate in the filtrate was quantified using Lactate Assay Kit-WST (Dojindo Laboratories, Kumamoto, Japan) according to the manufacturer’s protocol.

### 2.14 Western blot

Lung tissue homogenate described in lactate assay protocol was used for western blot analysis. Isolated cells or EVs were lysed in RIPA buffer (FUJIFILM) with protease inhibitor cocktail (Sigma), 40 mM sodium fluoride, and 2 mM sodium orthovanadate. Protein concentration of original samples was determined by the BCA or microBCA methods. Total proteins were separated by sodium dodecyl sulfate-polyacrylamide gel electrophoresis and transferred to polyvinylidene difluoride membranes. Membranes were incubated with the primary antibodies (**Table S1**). Samples were further incubated with horseradish peroxidase-linked antibodies (**Table S1**), followed by treatment of ECL™ Prime Western Blotting Detection Reagent (Cytiva, Marlborough, MA, USA). Protein bands were visualized using ImageQuant LAS 500 (Cytiva).

### 2.15 Quantification and statistical analysis

Statistical analysis was conducted using GraphPad Prism (version 5 and 9; GraphPad Software, San Diego, CA, USA). Results, except for survival analysis, were reported as mean ± standard deviation. Means were compared using the two-tailed unpaired Student’s *t*-test or the one-way ANOVA with the Turkey’s test. Survival curves were drawn using the Kaplan–Meier method and analyzed using the log-rank test. Values of P<0.05 were considered significant.

## 3 Results

### 3.1 Acidification and glycolytic reprogramming in pre-metastatic lungs of 4T1.2 syngeneic mouse model

**Figure 1:**
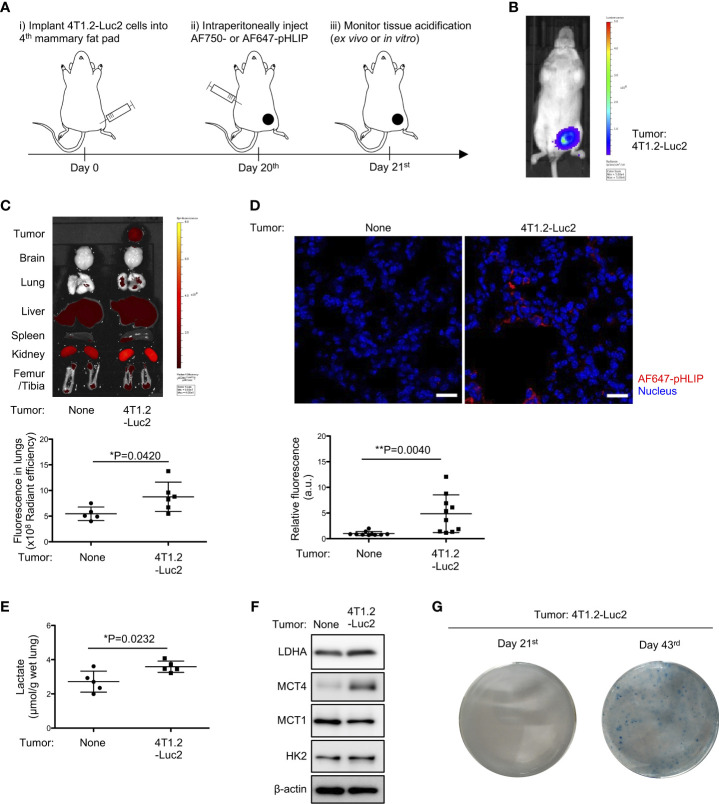
Notable accumulation of pHLIP and enhanced glycolysis in pre-metastatic lungs of mice inoculated with 4T1.2-Luc2 cancer cells. **(A)** Schematic presentation of mice treatment: i) BALB/c mice received vehicle or 4T1.2-Luc2 cancer cells (100,000 cells per fat pad). ii) On day 20^th^, mice received single injection of AF750- or AF647-pHLIP (2 nmol). iii) Next day (day 21^st^), animals were sacrificed. Fluorescence of AF750-pHLIP in harvested organs was recorded. **(B)** Bioluminescence image of mice at day 21^st^ after orthotopic implantation of 4T1.2-Luc2 cancer cells is shown. **(C)** *Ex vivo* fluorescence images of organs harvested from tumor-free mice (left) and 4T1.2-Luc2 tumor-bearing mice (right) are shown. The levels of fluorescent signal of pHLIP in lungs of tumor-free mice (5 animals) and 4T1.2-Luc2 tumor-bearing mice (6 animals) are presented. **(D)** PFA-fixed lungs of mice treated with AF647-pHLIP were stained with anti-Cy-5/AF647 antibody. Representative images of lungs harvested from tumor-free mice (left) and 4T1.2-Luc2 tumor-bearing mice (right) are shown. scale bars are 20 μm, and the levels of fluorescent signal of pHLIP in lung tissue slice per field are analyzed from each group (n = 10 fields) and shown. **(E)** The levels of lactate in lungs (5 animals per group) harvested at day 21^st^ from tumor-free mice and 4T1.2-Luc2 tumor-bearing mice are shown. **(F)** Western blot analyses of the indicated lysates from lungs harvested at day 21^st^ are presented. **(G)** Images of clonogenic metastatic colonies of 4T1.2-Luc2 cancer cells from lungs harvested at days 21^st^ and 43^rd^ after tumor cell injection into fat pad are shown. The data on graphs include all points, mean, and standard deviation. P-levels were calculated using two-tailed unpaired Student’s *t* test, *: P<0.05 and **: P<0.01.

To evaluate acidity in tissues/organs during pre-metastatic phase, we analyzed biodistribution of fluorescently labeled acidity marker, pHLIP, in orthotopically implanted syngeneic tumor model using *Luc2*-transduced cancer cell lines (**Figure 1A**). Firstly, 4T1.2-Luc2 breast cancer cells were inoculated into fourth mammary fat pad. Within 21 days, bioluminescence was observed in primary site only, where the cells were injected (**Figure 1B**), representing a pre-metastatic stage in the syngeneic tumor model. For biodistribution analysis, we used two fluorescent pHLIPs, where Alexa Fluor dyes were attached to the membrane non-inserting (N terminal) end of the peptide. Given the experimental suitability (19), AF750- and AF647-conjugated pHLIP agents were used for organ’s and tissue section’s imaging, respectively. We showed that AF750-pHLIP targeted acidic 4T1.2-Luc2 primary tumor and cleared via kidney, and fluorescent signal was also observed in liver, spleen, and femur/tibia of mice with and without tumor (**Figure 1C**). Image analyses of organs and tissue slices indicate that AF750-pHLIP was more significantly accumulated in lungs of 4T1.2-Luc2 tumor-bearing mice compared to the lungs extracted from the control mice without tumor (**Figures 1C, D**). Considering that tumor-generated lactic acid results in acidic tumor microenvironment (20), we measured lactate levels in the pre-metastatic lungs. Lactate levels were significantly increased in lungs of mice inoculated with 4T1.2-Luc2 cancer cells in mammary fat pad (**Figure 1E**). Notably, the expression of glycolysis-related proteins such as lactate dehydrogenase A (LDHA), monocarboxylate transporter 4 (MCT4), and hexokinase 2 (HK2) were also upregulated (**Figure 1F**). To investigate further the pre-metastatic stage, we assessed presence of micro-metastasis in lungs harvested at day 21^st^ and day 43^rd^ by clonogenic assay. 4T1.2 cell line was identified as a single cell clone of 4T1 which has resistance to 6-thioguanine (21). This property enabled to quantify metastasized cells in target organ by counting colonies grown in 6-thioguanine-containing medium (22). In clonogenic assay, no colony was yield from lungs harvested at day 21^st^ after cancer cell injection, while numerous colonies were observed in plating lungs harvested at day 43^rd^ (**Figure 1G**). Thus, the acidification and glycolytic enhancement occur during pre-metastatic phase, before 4T1.2-Luc2 cancer cells appear in lungs.

To investigate whether the acidification and metabolic reprograming were observed in another tumor syngeneic model, we inoculated 66cl4-Luc2 cancer cells into BALB/c mice. 66cl4 cell line is a subpopulation of 4T1 tumor with reduced metastatic capacity compared to 4T1.2 cells (16, 23, 24). After inoculation of 66cl4-Luc2 or 4T1.2-Luc2 cells (100,000 cells per animal), 66cl4-Luc2 tumor grew faster than 4T1.2-Luc2 tumor and reached larger sizes at day 21^st^ (**Figure S1A**). pHLIP fluorescence in lungs of mice inoculated with 66cl4-Luc2 breast cancer cells was lower compared to the fluorescence in lungs of control mice (**Figure S1B**). The lactate levels and expressions of glycolysis-associated proteins, except for monocarboxylate transporter 1 (MCT1), were not changed compared to the control (**Figures S1C-D**). These results suggest that onsets of acidification and glycolytic reprogramming in pre-metastatic lungs could be dependent on metastatic potential of inoculated cancer cells.

### 3.2 4T1.2 cell-derived secretome enhances accumulation of pHLIP and glycolysis in lungs

**Figure 2:**
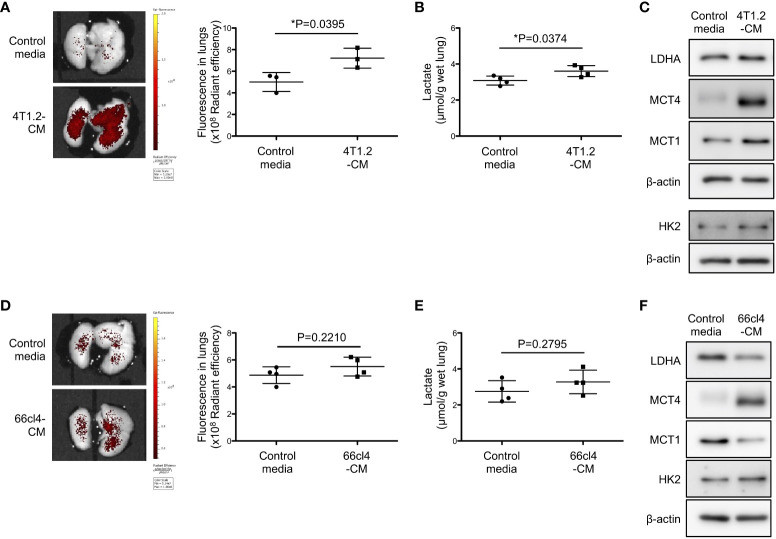
4T1.2 cancer cell-derived secretome enhances accumulation of pHLIP and glycolysis in lungs. **(A-F)** Tumor cell culture supernatant (conditioned media) or MEM-α (control media) were intraperitoneally injected once a day for 21 days. At the last day of medium treatment, mice were injected with AF750-pHLIP (2 nmol). The lungs were harvested and imaged at 24 h post injection of AF750-pHLIP. For other *in vitro* assays, lungs were harvested at 24 h after completion of 21 consecutive days of each medium administration. **(A)** Representative images of AF750-pHLIP fluorescence in lungs of mice treated with control media or 4T1.2-CM, and the levels of fluorescence (3 animals per group) are shown. **(B)** The levels of lactate in lungs (4 animals per group) harvested from mice treated with either control media or 4T1.2-CM are shown. **(C)** Western blot analyses of the indicated lysates from lungs of mice treated with control media or 4T1.2-CM are presented. **(D)** The representative images of AF750-pHLIP fluorescence in lungs of mice treated with control media or 66cl4-CM, and the levels of fluorescence (4 animals per group) are shown. **(E)** The levels of lactate in lungs (4 animals per group) of mice treated with control media or 66cl4-CM are shown. **(F)** Western blot analyses of the indicated lung lysates are presented. The data on graphs include all points, mean, and standard deviation. P-levels were calculated using two-tailed unpaired Student’s *t* test, *: P<0.05.

Focused on tumor cell secretome which is recognized to form pre-metastatic niche (25, 26), we studied the potential of 4T1.2 cancer cell-conditioned media (4T1.2-CM) to drive acidification and lactate overproduction in lungs. Aligned with the timeline of the previous experiments on tumor syngeneic models, we analyzed lungs of mice treated with 4T1.2-CM at day 21^st^. Consistent with the results obtained on 4T1.2-Luc2 tumor model, daily administration of 4T1.2-CM significantly increased pHLIP accumulation and lactate levels in lungs compared to lungs of mice that received control media (**Figures 2A, B**). Western blot analyses revealed that protein expressions associated with glycolysis (LDHA, MCT4, and MCT1) in lungs were also upregulated by 4T1.2-CM treatment (**Figure 2C**). Meanwhile, these tendencies were not reproduced in mice treated with 66cl4-CM (**Figures 2D-F**). Together, these results suggest that unique molecular component secreted by 4T1.2 cancer cells is responsible for acidification and enhancement of glycolysis in lungs during pre-metastatic phase.

### 3.3 Promotion of metastatic burden by lung acidification

**Figure 3:**
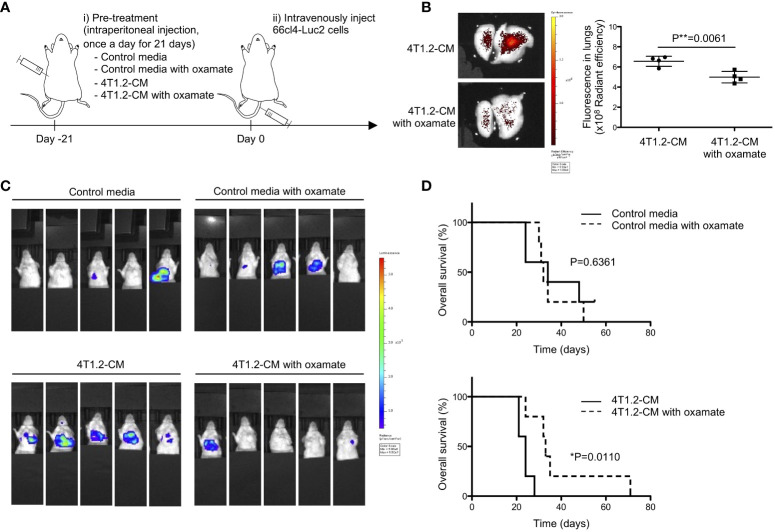
Lung acidification supports metastatic progression. **(A)** Schematic presentation of mice treatment: i) BALB/c mice received oxamate dissolved in either control media or 4T1.2-CM every day for 21 days. Mice treated with control media or 4T1.2-CM were prepared for control groups against oxamate-treated one. ii) At the day following the completion of pre-treatment (day 0), 66cl4-Luc2 cancer cells were injected into tail vein. **(B)** AF750-pHLIP (2 nmol) was injected at day 0. At 24 h post injection, mice were sacrificed to analyze pHLIP fluorescence in lungs. Representative image of *ex vivo* fluorescence in lungs harvested from mice treated with 4T1.2-CM (left) and oxamate-supplemented 4T1.2-CM (right), and the levels of fluorescence (4 animals per group) are shown. **(C)** Bioluminescent images of mice at day 21^st^ after intravenous injection of 66cl4-Luc2 cells are shown. Each group of mice received indicated pre-treatment before tumor cell injection. **(D)** Kaplan-Meier plots indicating survival time of mice (identical to ones in **C**) from intravenous injection of 66cl4-Luc2 cancer cells are presented (five animals were analyzed per group), and P-levels were calculated using log-rank test (*: P<0.05). The data on graph include all points, mean, and standard deviation, and P-level was calculated using two-tailed unpaired Student’s *t* test, **: P<0.01.

Prompted to the pro-metastatic effect of acidic pH in tumor microenvironment (27), we addressed the significance of lung acidification for metastasis formation. 66cl4-Luc2 cancer cells were injected into tail vein of mice to generate experimental lung metastasis following treatment of mice for 21 days by either MEM-α (control media) or 4T1.2-CM (**Figure 3A**). The results of 66cl4-CM treatment suggest that injected 66cl4-Luc2 cancer cells do not prime lung cells to cause acidification although intrinsic glycolysis of the injected cells could supply lactate for lung acidification during their metastatic growth. The LDHA inhibitor oxamate (28) was added to each medium to suppress lung acidification, which was evaluated by accumulation of AF750-pHLIP in lungs. Fluorescence of AF750-pHLIP dissolved in 4T1.2-CM was not significantly changed in a presence of oxamate (**Figure S2**), indicating that oxamate did not directly affect pHLIP fluorescence. *Ex vivo* analyses showed that pHLIP fluorescence was significantly diminished in lungs of mice treated with oxamate in 4T1.2-CM, compared to the lungs extracted from mice treated with 4T1.2-CM (**Figure 3B**). The obtained results indicate that lung acidification is related to lactic acid production by LDHA. At day 21^st^ post injection of 66cl4-Luc2 cancer cells, bioluminescence was detected only in 2 of 5 mice treated with 4T1.2-CM and oxamate, whereas it was observed in all (5 of 5) mice treated with 4T1.2-CM alone (**Figure 3C**). The rates of metastasis appearance in lungs detected by bioluminescence in mice treated with control media and control media/oxamate were 40% (2 of 5 mice) and 60% (3 of 5 mice), respectively. We plotted survival time of each mouse shown in **Figure 3C** as the time from 66cl4-Luc2 cancer cell injection (**Figure 3D**) and calculated median survival time (MST) for each group. 4T1.2-CM-administered mice had a MST of 24 days, and it was significantly prolonged by supplementation of oxamate (MST, 33 days). The MSTs for mice treated with control media and control media/oxamate were 34 and 32 days, respectively, and the difference was not found to be statistically significant. Not significant effect of oxamate added to control media treatment excludes direct action of injected 66cl4-Luc2 cancer cells. It suggests that acidified environment in lungs actively supports disseminated tumor cells for metastatic colonization.

### 3.4 pHLIP targets AT2 cells and some mature hematopoietic cells in acidic lungs

**Figure 4:**
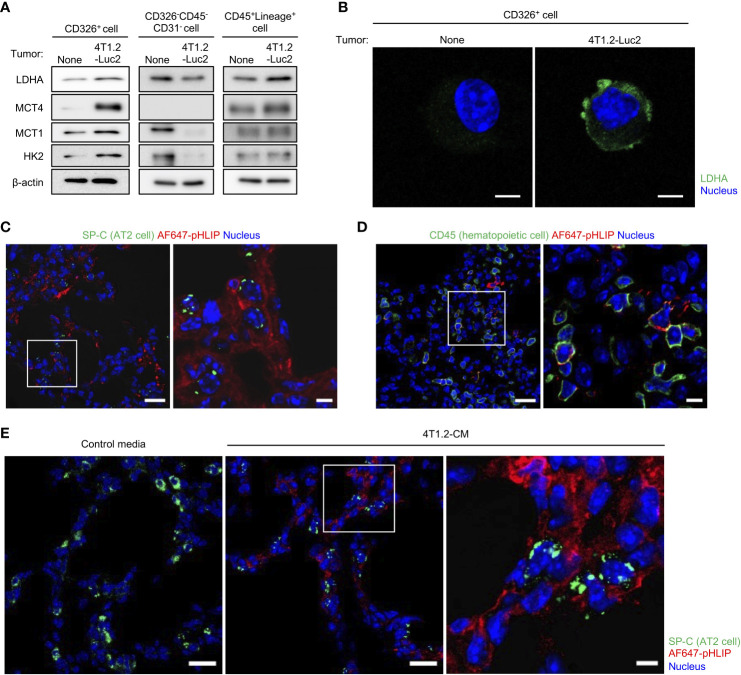
AT2 cells and mature hematopoietic cells as potential candidates to modulate acidity of pre-metastatic niche in lungs. **(A, B)** Live single lung cells were prepared from BALB/c mice harvested at day 21^st^ post injection of vehicle or 4T1.2-Luc2 cancer cells into 4^th^ mammary fat pad. After antigen staining, AT2 cells (CD326^+^), fibroblasts (CD326^-^CD45^-^CD31^-^) and mature hematopoietic cells (CD45^+^Lineage^+^) were sorted, followed by western blot analyses **(A)** and immunocytochemistry **(B)**. **(A)** Western blot analyses of the indicated lysates are shown. **(B)** Representative fluorescent images of lactate dehydrogenase A expression in AT2 cells is shown (scale bars are 5 μm). **(C, D)** Immunofluorescence analyses of pHLIP (red) and different stromal cell types (green) in lungs at day 21^st^ after orthotopic implantation of 4T1.2-Luc2 cells. AF647-pHLIP (2 nmol) was injected one day before harvest. Representative low-magnification images (left) and high-magnification images of the white-outlined areas (right) of PFA-fixed lung slices are shown. **(E)** Tissue localization of pHLIP in lungs of mice treated with either control media or 4T1.2-CM every day for 21 days is shown. At the last day of medium treatment, mice were injected with AF647-pHLIP (2 nmol) and sacrificed 24 h later. Representative low-magnification (left and middle) and high-magnification (right) images of PFA-fixed lung slices are shown. SP-C **(C, E)** and CD45 **(D)** are shown in green, AF647-pHLIP detected by anti-Cy5/AF647 antibody on the lung slices is shown in red, nucleus is shown in blue. Scale bars are 20 μm (left and middle) and 5 μm (right).

Considering the attenuation of pHLIP distribution in lungs by the LDHA inhibitor, we hypothesized that pHLIP targets glycolytic cells or their surrounding cells in lungs. To address this, we performed flow cytometry to analyze cells dissociated from lungs 24 h post injection of AF647-pHLIP into 4T1.2-Luc2 tumor-bearing mice. Cell population with pHLIP fluorescence was not detected, possibly due to proteolytic degradation of the agent in a process of tissue dissociation. For another approach, we performed cell sorting of three different cell types (epithelial cells, fibroblasts, and mature hematopoietic cells) from the lungs (**Figure S3**) and analyzed the expressions of glycolysis-related proteins in these cellular populations. The AT2 cell marker surfactant protein C (SP-C) was exclusively expressed in the CD326^+^ cells (**Figure S4**). No detection of luciferase-expressing cells indicated that metastasized 4T1.2-Luc2 cancer cells were not included in the sorted population. Notably, the CD326^+^ AT2 cells from the acidic lungs had a sharply high expressions of LDHA, compared to those from normal lungs (**Figures 4A, B**). The upregulation of glycolytic proteins was slightly observed in CD45^+^Lineage^+^ mature hematopoietic cells, but not in CD326^-^CD45^-^CD31^-^ fibroblasts. To confirm that the 2 cellular populations with enhanced glycolysis are targeted by pHLIP, we studied co-localization of fluorescent pHLIP with each cell type marker in lung tissue from 4T1.2 tumor-bearing mice. Immunohistochemical analyses revealed that pHLIP was largely localized in SP-C expressing cells although co-localization of pHLIP with CD45 was observed in a limited area (**Figures 4C, D**). AT2 cells targeted by pHLIP were also observed in lungs acidified by treatment of 4T1.2-CM (**Figure 4E**). Together, these results suggest potential role of glycolytic reprogramming in AT2 cells and some mature hematopoietic cells for lung acidification.

### 3.5 4T1.2-EVs induce acidification and glycolytic reprogramming in lungs

**Figure 5:**
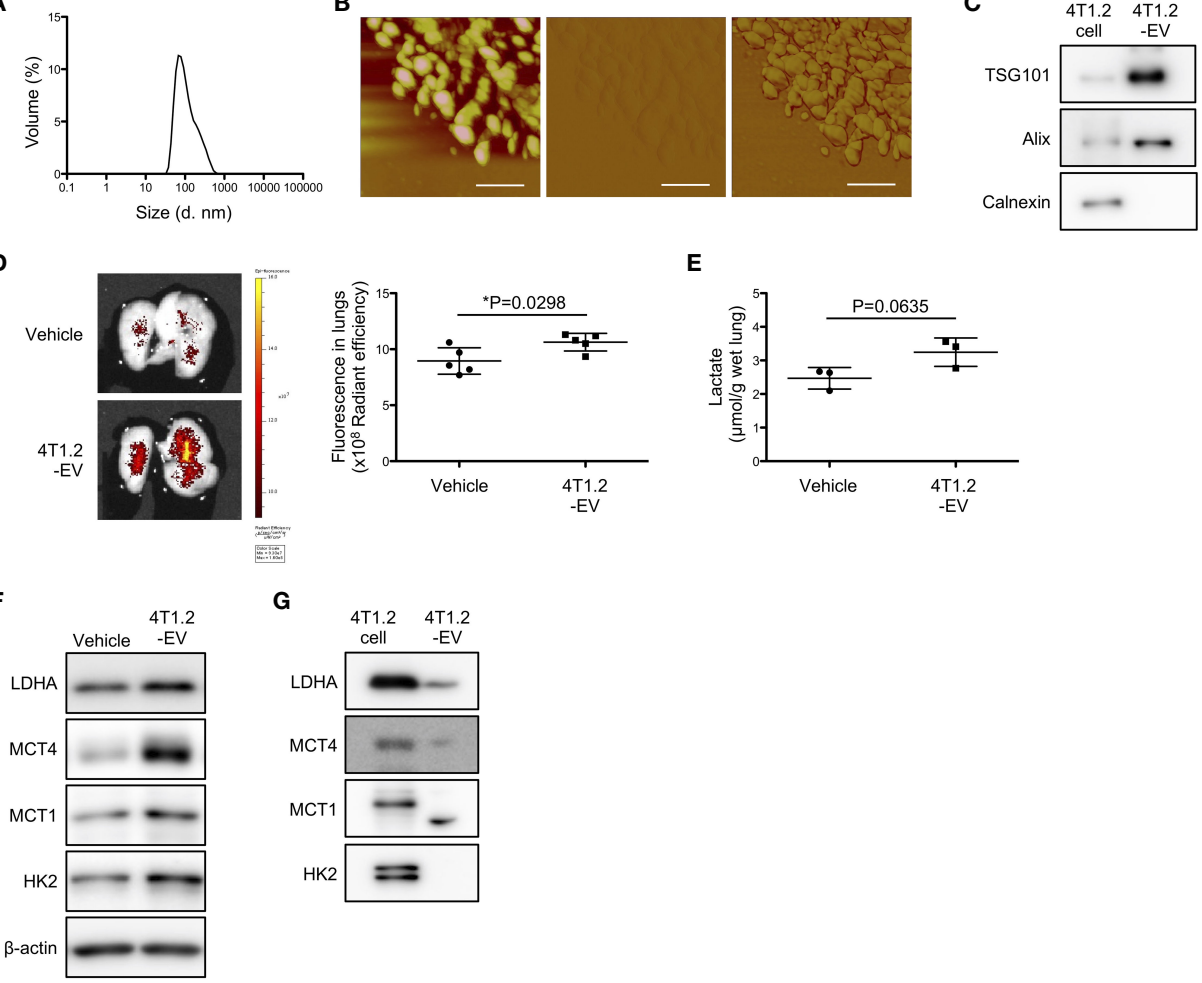
4T1.2-EVs induce extracellular acidosis and metabolic reprogramming in lungs. **(A-C, G)** The conditioned medium of 4T1.2 cancer cells was sequentially centrifuged at increasing centrifugal force, generating a 100,000 g pellet of 4T1.2-EVs. **(A)** Particle size distribution of 4T1.2-EVs measured by DLS is shown. **(B)** Representative topographic (left), amplitude (middle), and phase (right) images of 4T1.2-EVs analyzed by atomic force microscopy are presented (scale bars are 200 nm). **(C)** Western blot analyses of 4T1.2-EV for EV-enriched protein expression is presented. **(D-F)** BALB/c mice received 10 μg of 4T1.2-EVs or vehicle every day for 21 days through a peritoneal route. At the last day of 4T1.2-EV treatment, mice were injected with AF750-pHLIP (2 nmol), and harvested lungs were imaged at 24 h post injection. For other *in vitro* assays, lungs were harvested at 24 h after completion of 21 constitutive days administration of 4T1.2-EVs. **(D)** Representative images of AF750-pHLIP fluorescence in lungs of mice treated with vehicle or 4T1.2-EVs, and the levels of fluorescence (5 animals per group) are shown. **(E)** The levels of lactate in lungs (3 animals per group) from mice harvested from vehicle- or EV-treated mice are shown. **(F)** Western blot analyses of the indicated lysates from lungs of mice treated with vehicle or 4T1.2-EVs is presented. **(G)** Expressions of glycolysis-related proteins in 4T1.2-EV were analyzed by western blot and presented as images of protein band. The data on graphs include all points, mean, and standard deviation, and P-levels were calculated using two-tailed unpaired Student’s *t* test, *: P<0.05.

EV-mediated pre-metastatic niche formation is widely reported in several tumor types including 4T1 tumor (26, 29, 30), which encouraged us to assess whether 4T1.2-EVs can induce lung acidification. Firstly, we purified EVs from 4T1.2-CM by ultracentrifugation. DLS analysis of 100,000 g pellet (4T1.2-EVs) showed a single population peaked at 100 nm (**Figure 5A**). Concordant results were observed in atomic force microscopy analysis that size and nanostructure of 4T1.2-EVs were measured by acquiring topographic (height), amplitude, and phase images (**Figure 5B**). This indicates that 4T1.2-EVs are approximately 100 nm in diameter and non-uniform in shape. In western blot analyses, the EV markers (CD63 and TSG101) were detected in 4T1.2-EVs, whereas the endoplasmic reticulum marker, Calnexin, was not detected (**Figure 5C**). This proved little or no contamination of 4T1.2-EVs from cellular organelles. Notably, the secreted amounts of protein in EVs collected from 4T1.2 cells was significantly higher than the amounts of protein in collected EVs from 66cl4 cells (**Figure S5**). To examine the potential of 4T1.2-EVs for lung acidification, we peritoneally administered 4T1.2-EVs to naïve mice daily for 21 days, followed by evaluation of pHLIP targeting and lactate production in lungs. pHLIP fluorescence was significantly higher in lungs of mice treated with 4T1.2-EVs compared to lungs extracted from mice treated with vehicle (**Figure 5D**). Notably, the EV treatment also increased lactate amounts (**Figure 5E**) and expressions of glycolysis-related proteins (**Figure 5F**) in lungs. Given that EVs reportedly transport functional proteins associated with glucose metabolism (31), we analyzed expression levels of glycolysis-associated proteins in 4T1.2-EVs using western blot. In 4T1.2-EV lysate, LDHA and MCT4 were detected as similar sized bands as in 4T1.2 cancer cell lysate (**Figure 5G**). Smaller sized protein band probed by anti-MCT1 antibody was observed in 4T1.2-EVs, compared to 4T1.2 cancer cells. This might be a proteolytic form of MCT1 as previously described by Merezhinskaya et al. (32). The obtained results suggest that 4T1.2-EVs harboring glycolytic proteins are an inducer for acidification and enhancement of glycolysis in lungs.

### 3.6 pHLIP-Dex suppresses metastasis by regulating inflammatory response in acidic lungs

**Figure 6:**
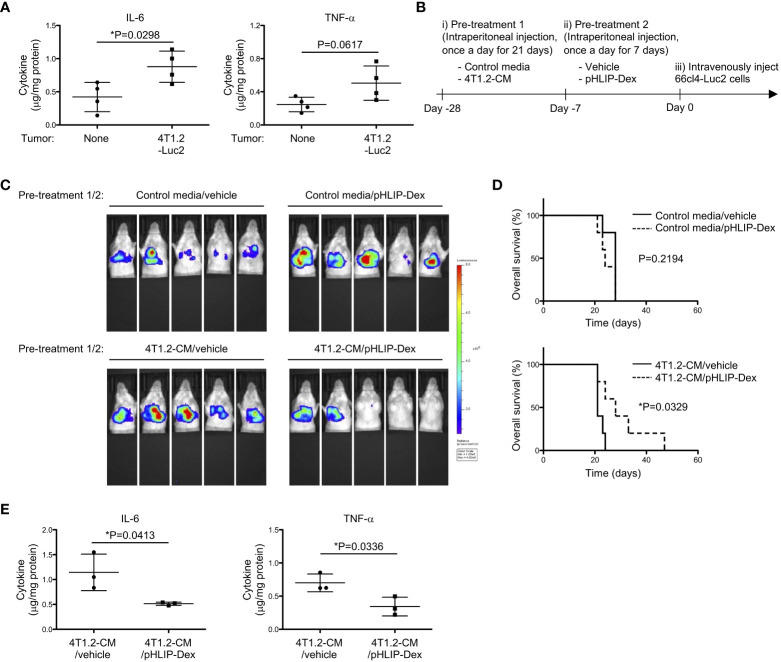
Anti-metastatic effect of pHLIP-Dex targeting acidic niche in lungs. **(A)** The levels of inflammatory cytokines in lung tissues from BALB/c mice (4 animals per group) harvested at 21^st^ post orthotopic injection of vehicle or 4T1.2-Luc2 cancer cells analyzed by cytometric bead assay are shown. **(B)** Schematic presentation of mice treatment: i) BALB/c mice were treated with either control media or 4T1.2-CM every day for 21 days (10 animals per group). ii) Each group was further split into 2 groups to treat with vehicle or pHLIP-Dex (4 mg/kg) by intraperitoneal injection once a day for the next 7 days (4 groups with 5 animals/group). iii) At the day following the completion of the second pre-treatment (day 0), 66cl4-Luc2 cancer cells were injected into tail vein. **(C)** Bioluminescent images of mice at day 17^th^ after intravenous injection of 66cl4-Luc2 cancer cells are shown. Each group of mice received indicated pre-treatments before tumor cell injection. **(D)** Kaplan-Meier plots indicating survival time of mice (identical to ones in **C**) from intravenous injection of 66cl4-Luc2 cancer cells are presented (5 animals were analyzed per group). P-levels were calculated using log-rank test (*: P<0.05). **(E)** The levels of inflammatory cytokines in lung tissues from BALB/c mice, which received indicated pre-treatments, are shown. Lungs were harvested at day 0 without tumor cell injection. Data analyzed by cytometric bead assay (3 animals per group) were shown. The data on graphs include all points, mean, and standard deviation, and P-levels were calculated using two-tailed unpaired Student’s *t* test, *: P<0.05.

In line with strong association between glycolysis and pro-inflammatory response (30, 33), we evaluated inflammation levels of acidified lungs by measuring pro-inflammatory cytokines. Notably, levels of IL-6 and TNF-α were increased in lungs from 4T1.2-Luc2 tumor-bearing mice (**Figure 6A**). Lungs of mice treated with 4T1.2-CM also showed significant increase of IL-6 levels compared to lungs of MEM-α, whereas TNF-α levels were not increased in lungs of 4T1.2-CM-treated mice. Supplementation of oxamate did not influence the IL-6 levels elevated by treatment of 4T1.2-CM (**Figure S6**). Focused on a supporting role of pro-inflammatory cytokines in metastasis, we addressed a potential therapeutic target of the inflammation for development of lung metastasis. The accumulation of pHLIP in the inflamed pre-metastatic lungs suggested potential utilization of pHLIP-drug conjugates as targeted therapeutics for enhancement of therapeutic efficacy. Therefore, we synthesized pHLIP conjugated with potent anti-inflammatory drug, dexamethasone, (pHLIP-Dex). Dexamethasone was conjugated to the membrane-inserting C-terminal end of the peptide via a self-immolating disulfide cleavable linker, the strategy which was implemented previously (34). After medium treatment, we administered pHLIP-Dex for 7 days and subsequently injected 66cl4-Luc2 cancer cells into tail vein (**Figure 6B**). To evaluate metastatic burden of all mice used in the therapeutic study, the bioluminescent imaging at Day 17 was performed (**Figure 6C**). In control media-treated groups that received either vehicle or pHLIP-Dex, all (10 of 10) mice showed bioluminescence signal in their thorax. In the group that received control media and pHLIP-Dex, the signal seems to be slightly enhanced, compared to that in control media/vehicle group. However, Kaplan-Meier survival plot demonstrated no significant effect of pHLIP-Dex on survival of mice received pre-treatment of control media (**Figure 6D**); the MSTs for mice treated with control media/vehicle and control media/pHLIP-Dex were 28 and 24 days, respectively. In contrast to the results obtained on control media-treated mice, remarkable delay of metastatic growth was observed when pHLIP-Dex was administered to mice that received pre-treatment of 4T1.2-CM. In 4T1.2-CM/pHLIP-Dex group, 3 out of 5 mice did not show any bioluminescent signal, although all (5 of 5) mice exhibited high bioluminescence in 4T1.2-CM/vehicle group. The MST for mice treated with 4T1.2-CM/pHLIP-Dex (28 days) was longer than that for 4T1.2-CM/vehicle (21 days). Statical analysis revealed statistically significant difference of mice survival between the groups of 4T1.2-CM/vehicle and 4T1.2-CM/pHLIP-Dex. Notably, levels of IL-6 and TNF-α in lungs were significantly decreased by pHLIP-Dex administration (**Figure 6E**). Together, the obtained results suggest that pHLIP-Dex acts on the acidic lungs to diminish inflammation during pre-metastatic stage, which results in prevention of metastatic progression.

## 4 Discussion

Metastasis is reportedly the primary cause of death from solid tumors, which is explained by the lack of adequate control of development of tumor metastases (35). Disseminated tumor cells are resistant to a variety of current therapies, and some chemotherapeutic drugs even exhibit metastasis-promoting effect (36). Accumulated evidence has deepened understanding of biological processes of metastasis, which allows the development of technologies for diagnosis and therapy of metastasis (37–39). Especially, pre-metastatic niche is of great interest to both researchers and health professionals. The goal is to detect pre-metastatic niche and therapeutically interfere with the progression of metastases. Aligned with this, we report the first demonstration of acidic lung pre-metastatic niche targeted by pHLIP, which is created by injection of tumor cell-derived EVs.

Since the report in 2006 by Reshetnyak et al. (40), numerous basic studies have demonstrated targeting of acidic disease tissue by pHLIP. Utility of pHLIP as a pH responsive probe has been established particularly in oncology research. Protonated by extracellular acidity of tumor, pHLIP effectively inserts across membrane of acidic cells and thus stably and preferentially retained in tumor. Imaging agents conjugated with pHLIP allows for tumor visualization and has been translated into clinical trials (41). In contrast to these tumor imaging studies, here we applied pHLIP technology to image acid-base status of pre-metastatic niche that was created in a tumor-free organ. Like tumor microenvironment, pre-metastatic niche consists of various cellular components including endothelial cells, fibroblasts, immune cells, etc. The mechanisms by which these cells contribute to formation of tumor supportive environment in pre-metastatic niche is partly compatible with the role of these cells in primary tumor microenvironment; therefore, pre-metastatic niche might share features of primary tumor site. Considering the hypothesis, we were focused on extracellular acidosis, a well-characterized feature of tumor microenvironment, and its metastasis-promoting potential. *Ex vivo* pHLIP imaging revealed the significant acidification of pre-metastatic lungs from mice orthotopically inoculated with 4T1.2-Luc2 cancer cells or treated with 4T1.2 cancer cell-conditioned media. Also, lactate amount and expression of proteins associated with glucose metabolism were increased in the lungs. When mice were treated with the LDHA inhibitor oxamate upon administration of 4T1.2-CM, the lung acidification was suppressed. Thus, pHLIP detects the acidification associated with glycolytic reprogramming in pre-metastatic lungs. Positron emission tomography imaging with ^89^Zr-labelled pHLIP, which is currently in translation to clinics (42), could be used for imaging of pre-metastatic niche in humans.

Pre-treatment of oxamate suppressed lung metastatic burden of intravenously injected 66cl4-Luc2 cancer cells, implying that the high extracellular acidity in lungs is a supportive condition for colonization of disseminated tumor cells. In previous reports (43, 44), buffer bicarbonate therapy was used to neutralize tumor acidity and suppressed spontaneous lung metastasis. This prompted us to ask whether drinking bicarbonate buffer influenced metastatic development via attenuating acidification of pre-metastatic lung. We served BALB/c mice 0.2 M sodium bicarbonate *ad libitum* during 21 days of medium treatment. We observed that the intake of drinking liquid was significantly reduced in these mice, and they lost body weight (data not shown). Difference of taste response and ingestive behavior among mouse strains might be a reason why the buffer therapy was not tolerable in our model although the effect of bicarbonate buffer on animal weight was not described in the study by Pilon-Thomas et al. (44). Considering the experimental limitation in directly modifying local acidity in our model, we attempted to cut off a source of acid by inhibiting glycolysis pathway. Inhibition of LDHA by oxamate reportedly induced G2/M arrest/apoptosis and attenuated *in vivo* tumor growth of nasopharyngeal carcinoma (45), suggesting a mechanism by which oxamate acts on tumor cells. In our study, direct action of oxamate on tumor cells was ruled out as its anti-metastatic effect was observed when it was combined with 4T1.2-CM treatment, but not with control media treatment. Lung metastasis seems to develop more rapidly in 4T1.2-CM treated mice than in mice with MEM-α treatment. IL-6 increased in 4T1.2-CM-primed lungs may be a factor for increased metastasis. This possibility is supported by Chang et al., who showed that IL-6 derived from invasive breast cancer cells regulated expansion of myeloid-derived suppressor cells and macrophage infiltration in distant organs (46). Meanwhile, the elevated IL-6 levels were not significantly changed by oxamate, suggesting no association between the anti-metastatic effect of oxamate and IL-6 levels in lungs. Using a preclinical research model for non-small cell lung cancer, Qiao et al. revealed that oxamate increased the infiltration of activated CD8^+^ T cells in the tumor and thus enhanced the therapeutic effect of anti-programmed cell death 1 antibody (47). This insight will prompt us to investigate whether activated CD8^+^ T cells mediates the effect of oxamate to suppress metastatic colonization of 66cl4-Luc2 cancer cells in lungs.

Tumor acidification is thought to be mainly resulting from lactate overproduction by highly proliferative tumor cells which utilize glycolysis independent of oxygen availability. Meanwhile, some types of stromal cells in tumor undergo metabolic shift toward glycolysis which is associated with their pro-tumorigenic functions (48, 49). In line with the findings about glycolysis reprogramming in non-cancerous host stromal cells, we demonstrated the upregulated glucose metabolism and localization of pHLIP in AT2 cells and some CD45^+^ hematopoietic cells which may contribute to formation of the acidic environment by supplying the acidic metabolite, lactate. Our findings also suggest that 4T1.2 cancer cells utilize EVs to cause reprogramming of glucose metabolism and acidification in lungs potentially by transferring glycolysis-related proteins. The secreted amounts of EV-loaded proteins from 4T1.2 cells were approximately 100-fold higher than those from 66cl4 cells. The results are in a good agreement with the previous study, which showed that 66cl4 cells secreted less numbers of EVs loading lower amounts of proteins compared to 4T1.2 cells (50). The remarkable difference of EV protein yield may explain that 4T1.2 cells but not 66cl4 cells create the acidic pre-metastatic niche in lungs. Given our proposed mechanism, molecular composition of EVs is also a critical aspect to determine whether their parental cancer cells induce lung acidification. Glycolytic proteins were identified in EVs from human cancer cell lines of colorectal and prostate cancers (51, 52). Dong et al. demonstrated that tumor LDHA expression and serum LDH levels are associated with brain metastasis of triple negative breast cancer (53). The evidence speculates that our concept can be applicable into clinical practice.

AT2 cells are crucially involved in lung homeostasis through production of pulmonary surfactant to support gas exchange and, as a progenitor cells, to renewal of alveolar epithelial cells. Additionally, AT2 cells participate in innate immune response of lung by releasing several cytokines and chemokines. Tumor exosomal RNAs promote the chemokine secretion through activation of their Toll Like Receptor 3 on AT2 cells, which recruits neutrophils for lung pre-metastatic niche formation (54). Immunosuppressive tumor microenvironment contains metabolically active cancer-associated fibroblasts and tumor-associated macrophages which were targeted by pHLIP as described previously (34). This suggests that glycolytic macrophages may be the pHLIP-targeted mature hematopoietic cells in the acidic lungs. It warrants more detailed investigation to address the potential action of 4T1.2-EVs in glycolytic reprogramming of AT2 cells and the subset of CD45 cells targeted by pHLIP.

Our findings indicate the presence of inflammation in the pHLIP-targeted pre-metastatic lungs. This is supported by the fact that extracellular acidification is long known to be raised in inflamed tissue (55). Several inflammatory disease models showed that pHLIP targeted inflamed sites (56, 57). pHLIP technology offers new opportunity to develop targeted therapy of acidic diseased tissues. In recent study reported by Moshnikova et al. (34), the agonist of stimulator of interferon genes (STINGa) was targeted to acidic tumor stroma by pHLIP and elicited an effective anti-tumor immune response. In the present study, we introduced pHLIP-targeted steroid, dexamethasone, (pHLIP-Dex) as a potent therapeutic approach to prevent metastatic development by suppressing inflammation in the lungs with acidic pre-metastatic niche. Pro-inflammatory cytokines in pre-metastatic niche recruit bone marrow-derived cells for development of an immunosuppressive microenvironment (58), which may be blocked by pHLIP-Dex.

We conclude that pHLIP detects the extracellular acidosis in lungs, resulting from metabolic priming of pulmonary cells by tumor cell-secreted extracellular vesicles. Combined with the pHLIP technology, the therapeutic cargo dexamethasone was delivered to the acidic niche, attenuating metastasis development effectively. The study sheds light on the mechanism of pre-metastatic niche formation and provides insights in design of novel diagnostic and therapeutic approaches for tumor metastasis.

## Data availability statement

The raw data supporting the conclusions of this article will be made available by the authors, without undue reservation.

## Ethics statement

The animal study was approved by the Ethics of Animal Research of Kyoto Pharmaceutical University. The study was conducted in accordance with the local legislation and institutional requirements.

## Author contributions

TM: Data curation, Investigation, Validation, Writing – original draft, Writing – review & editing, Conceptualization, Formal Analysis, Methodology, Project administration, Visualization. YT: Conceptualization, Funding acquisition, Investigation, Project administration, Writing – original draft, Writing – review & editing, Methodology, Supervision, Visualization. HS: Data curation, Investigation, Validation, Writing – review & editing. RI: Data curation, Investigation, Validation, Writing – review & editing. KK: Data curation, Investigation, Validation, Writing – review & editing. AM: Resources, Writing – review & editing, Investigation. OA: Funding acquisition, Resources, Writing – review & editing, Investigation. SH: Conceptualization, Writing – review & editing, Methodology. YR: Conceptualization, Funding acquisition, Resources, Supervision, Writing – original draft, Writing – review & editing, Investigation. EA: Conceptualization, Supervision, Writing – review & editing.
