# Supplementary material for: Targeting acidic pre-metastatic niche in lungs by pH low insertion peptide and its utility for anti-metastatic therapy
Source: Front Oncol. 2023 Nov 15;13:1258442. doi: 10.3389/fonc.2023.1258442 (PMC10684925; doi:10.3389/fonc.2023.1258442)
Supplement: Supplementary file 1 [file DataSheet_1.pdf]

Supplementary Material

Targeting acidic pre-metastatic niche in lungs by pH Low Insertion Peptide and its utility for anti-metastatic therapy

Toma Matsui[1], Yuki Toda[1]*, Haruka Sato[1], Rina Itagaki[1], Kazuya Konishi[1], Anna Moshnikova[2], Oleg A. Andreev[2], Shigekuni Hosogi[1], Yana K. Reshetnyak[2], Eishi Ashihara[1]

***Correspondence:** Yuki Toda: tda@mb.kyoto-phu.ac.jp

Table S1. Primary and secondary antibodies used in this study.

**Primary**			
**Product name**	**Clone**	**Use (dilution)**	**Source (Identifier)**
Rabbit anti-LDHA	N/A	WB (1:1000), ICC (1:1000)	Cell Signaling Technology (2012)
Mouse anti-MCT4	D-1	WB (1:500)	Santa Cruz (sc-376140)
Rabbit anti-MCT1	N/A	WB (1:400)	Novus Biologicals (NBP1-59656)
Mouse anti-HK2	1A7	WB (1:1000)	Santa Cruz (sc-130358)
Mouse anti-β-actin	AC-15	WB (1:10000)	Sigma-Aldrich (A5441)
Mouse anti-TSG101	4A10	WB (1:1000)	Abcam (ab83)
Goat anti-Alix	N/A	WB (1:1000)	Santa Cruz (sc-49268)
Rabbit anti-Calnexin	N/A	WB (1:1000)	Abcam (ab22595)
Rat anti-mouse CD326, Biotin	G8.8	FCM (1:500)	Biolegend (118203)
Rat anti-mouse CD45, FITC	30-F11	FCM (1:500), IHC (1:1000)	BD Biosciences (553080)
Rat anti-mouse CD31, PE/Cy7	390	FCM (1:500)	eBioscience (25-0311-81)
Lineage Cell Detection Cocktail, Biotin	N/A	FCM (1:200)	Miltenyi Biotec (130-092-613)
Rat anti-mouse CD64, Biotin	X54-5/7.1	FCM (1:500)	Biolegend (139318)
Rat IgG2a, κ Isotype Control, PE/Cy7	N/A	FCM (1:500)	Biolegend (400521)
Rat IgG2a κ Isotype Control, Biotin	ES26-15B7.3	FCM (1:200-500)	Miltenyi Biotec (130-101-969)
Rat anti-mouse CD16/32	93	FCM (1:1000)	eBioscience (101302)

Goat anti-Luciferase	N/A	ICC (1:50)	Promega (G7451)
Rabbit anti-SP-C	N/A	IHC (1:1000), ICC (1:1000)	Merck Millipore (ABC99)
Mouse anti-Cy5	CY5-15	IHC (1:500)	Sigma-Aldrich (C1117)
**Secondary**			
Goat anti-rabbit IgG, HRP	N/A	WB (1:1000)	Cell Signaling Technology (7074)
Horse anti-mouse IgG, HRP	N/A	WB (1:1000-5000)	Cell Signaling Technology (7076)
Donkey anti-goat IgG, HRP	N/A	WB (1:1000)	Santa Cruz (sc-2020)
Mouse anti-Biotin, AF647	N/A	FCM (1:500)	Jackson ImmunoResearch (200-602-211)
Donkey anti-mouse IgG (H+L), AF647	N/A	IHC (1:500), ICC (1:200)	Jackson ImmunoResearch (715-605-151)
Goat Anti-rat IgG (H+L), AF488	N/A	IHC (1:500)	Jackson ImmunoResearch (112-545-167)
Goat anti-rabbit IgG (H+L), AF488	N/A	IHC (1:500), ICC (1:500)	Invitrogen (A-11008)
Donkey anti-goat IgG (H+L), AF488	N/A	ICC (1:500)	Invitrogen (A-11055)

Abbreviations: FCM, flow cytometry; ICC, immunocytochemistry; IHC, immunohistochemistry; N/A, not applicable; WB, western blot

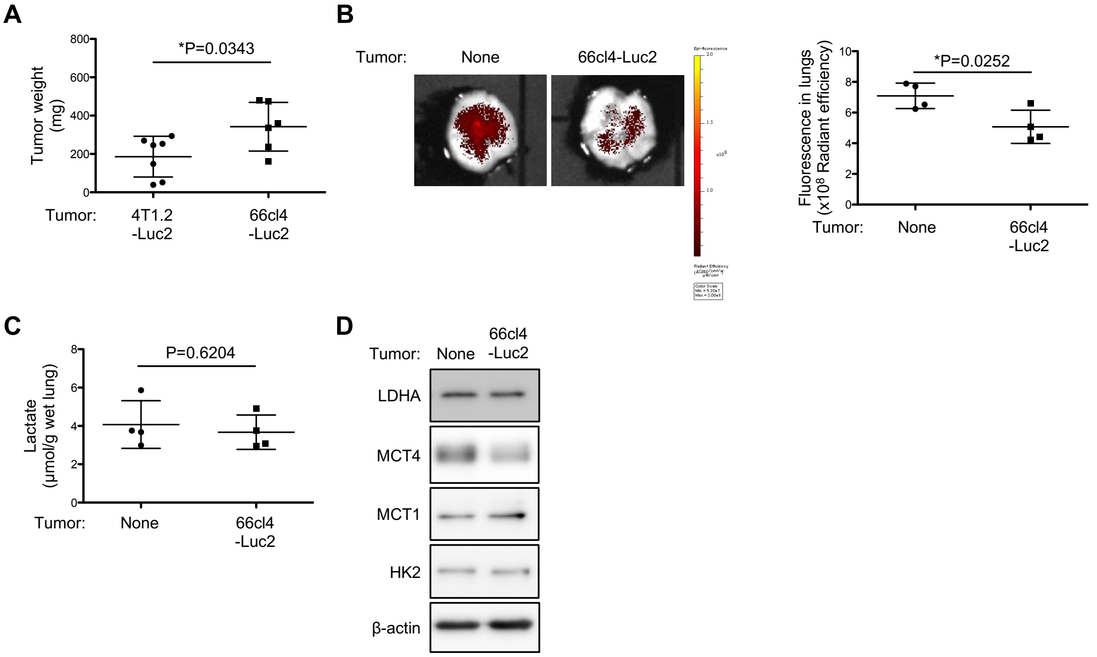

Figure S1. Biodistribution of pHLIP and lactate metabolism in lungs of mice orthotopically inoculated with 66cl4-Luc2 cancer cells. (A-D) 66cl4-Luc2 (100,000) cells were injected into 4th mammary fat pad. Single intraperitoneal injection (2 nmol) of AF750-pHLIP was received at day 20th after tumor cells injection. Analysis of harvested lungs by *in vivo* imaging was performed at 24 h post injection of AF750-pHLIP. **(A)** Weight of 66cl4-Luc2 (7 animals) and 4T1.2-Luc2 (6 animals) tumors at day 21st after orthotopic implantation is shown. **(B)** Representative images of AF750-pHLIP fluorescence in lungs of mice at 21st day after orthotopic transplantation of 66cl4-Luc2 cells. Four animals were analyzed from each group. **(C)** Levels of lactate in lungs (4 animals per group) harvested at 21st day after 66cl4-Luc2 cell injection in comparison with the level of lactate in lungs from control mice without tumor. **(D)** Western blot analyses of the indicated lysates from lungs harvested at day 21st after tumor cell injection. All presented data include all points, means, and standard deviations. P-levels were calculated using two-tailed unpaired Student's *t* test (*: $P<0.05$).

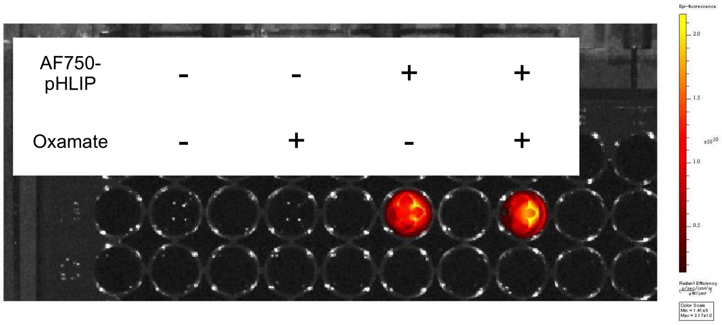

Figure S2. Fluorescent image of 4T1.2-CM in 96 well plate is shown. Each sample contained 20 μM AF750-pHLIP or 20 mg/mL oxamate.

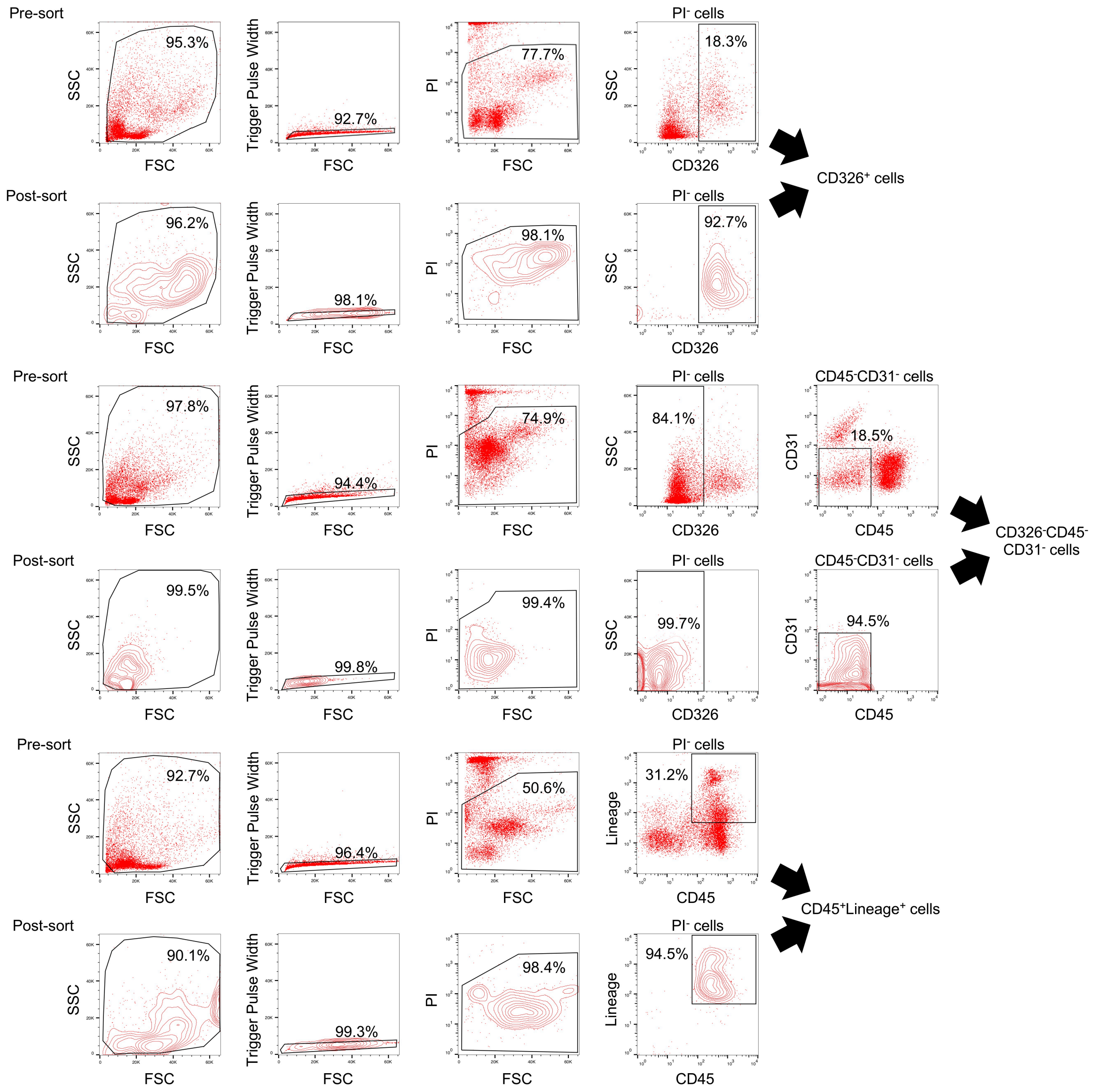

Figure S3. Flow cytometric plots showing gating strategy for 3 different cells from lungs

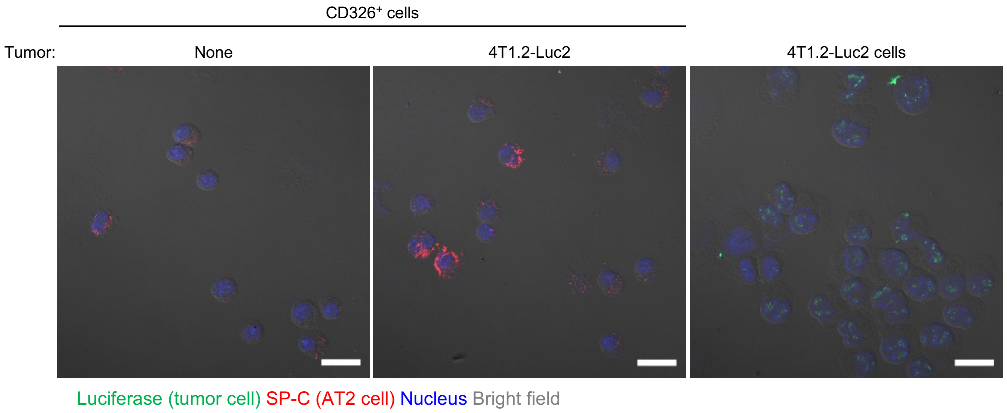

Figure S4. Identification of sorted CD326^{+} AT2 cells Representative fluorescent images of SP-C (red) and Luciferase (green) in CD326 cells sorted from lungs of tumor-free mice (left) or 4T1.2-Luc2 tumor-bearing mice (middle) were shown. 4T1.2-Luc2 cells were a positive control for analysis of luciferase expression (right). Scale bars were 20 μm.

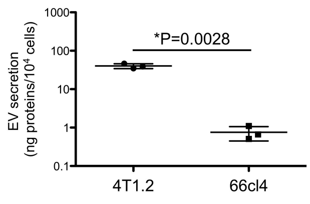

Figure S5. EV secretion capacity of 4T1.2 and 66cl4 cells. EV protein levels collected from culture supernatant of each cancer cells (3 samples per group). All presented data include all points, means, and standard deviations. P-levels were calculated using two-tailed unpaired Student's *t* test (*: P<0.05).

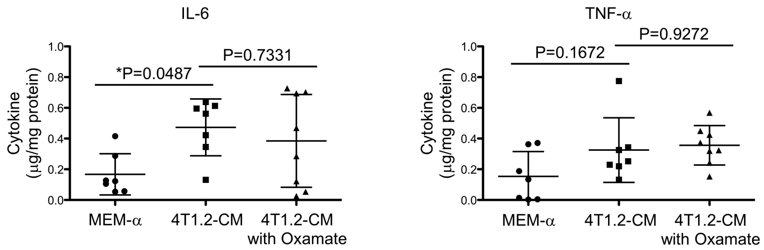

Figure S6. Inflammatory state of lungs in medium-treated mice. The levels of inflammatory cytokines in lung tissues from BALB/c mice harvested from mice which received 21 constitutive days administration of either MEM-α (7 animals), 4T1.2-CM (7 animals), or oxamate-supplemented 4T1.2-CM (8 animals). Lungs were harvested at the following day to complete the indicated treatments. All presented data include all points, means, and standard deviations. P-levels were calculated using one-way ANOVA with the Turkey's test (*: $P<0.05$).
